# Therapeutic Role of Probiotics for the Treatment of Dyspepsia: A Review of the Literature

**DOI:** 10.1111/nmo.70057

**Published:** 2025-05-07

**Authors:** Giovanni Marasco, Miriam Fiocca, Cesare Cremon, Luigi Colecchia, Marcello Maida, Elton Dajti, Maria Raffaella Barbaro, Vincenzo Stanghellini, Giovanni Barbara

**Affiliations:** ^1^ IRCCS Azienda Ospedaliero Universitaria di Bologna Bologna Italy; ^2^ Department of Medical and Surgical Sciences University of Bologna Bologna Italy; ^3^ Department of Medicine and Surgery University of Enna ‘Kore’ Enna Italy

**Keywords:** Bifidobacterium, functional dyspepsia, *Helicobacter pylori*, lactobacillus, probiotic, *Saccharomyces boulardii*, supplementation

## Abstract

**Background:**

Dyspepsia is a common condition with a high prevalence in the general population. Patients in whom traditional diagnostic procedures can detect no identifiable explanation for the symptoms are diagnosed as being affected by functional dyspepsia (FD). To date, no etiological therapy for FD is available, and the current management includes general measures, acid‐suppressive drugs, prokinetic agents, fundus‐relaxing drugs, antidepressants, and psychological interventions. Recent evidence suggests that microbiota imbalance is involved in the development of FD. As a consequence, the modulation of microbiota through the use of probiotics could represent an effective therapeutic strategy. Moreover, *Helicobacter pylori* (HP) infection is a frequent cause of dyspepsia, and patients diagnosed with HP‐associated dyspepsia are treated with *HP* eradication. In this regard, probiotics supplementation may also be helpful for HP infection to increase the eradication success rate as well as to reduce gastrointestinal adverse events caused by antibiotics.

**Purpose:**

This review of the literature aims to summarize and discuss the current evidence on the use of probiotics in the treatment of dyspepsia and as a supplement to HP eradication therapy.

## Graphical abstract

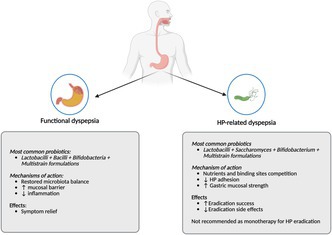

**Summary**

- Recent studies suggest that microbiota imbalance plays a significant role in the development of functional dyspepsia (FD).
- Specifically, patients with FD have shown increased levels of Streptococcus and decreased levels of Prevotella, Veillonella, and Actinomyces.
- Several clinical trials have reported promising efficacy of probiotics in restoring microbiota balance and alleviating FD symptoms.
- Additionally, probiotics may enhance Helicobacter pylori (HP) eradication rates and reduce gastrointestinal side effects associated with antibiotics.
- Meta‐analyses indicate that specific probiotic strains, such as Lactobacillus and Bifidobacterium, improve eradication success and patient compliance.

## 1 Introduction

Dyspepsia is defined according to the Rome IV criteria as any combination of the following 4 symptoms: postprandial fullness, early satiety, epigastric pain, and epigastric burning that are severe enough to interfere with the usual activities and occur at least 3 days per week over the last 3 months with an onset of at least 6 months in advance [1]. Dyspepsia can be initially classified into three groups depending on whether upper gastrointestinal endoscopy has been performed and, if so, whether relevant pathology is detected: uninvestigated dyspepsia, organic dyspepsia, and functional dyspepsia (FD). After an accurate history taking and physical exam, in the absence of alarm symptoms and signs, patients are diagnosed as being affected by uninvestigated dyspepsia and can be treated empirically on the basis of their clinical manifestations [1]. Uninvestigated dyspepsia prevalence varies between countries and ranges from 17.6% in studies defining uninvestigated dyspepsia according to Rome I criteria to 6.9% in those using Rome IV criteria [2]. If the response is unsatisfactory or early relapses occur, a test and treat approach for *Helicobacter pylori* (*HP*) infection is recommended [3]. A recent systematic review estimated that more than 4 billion individuals would be positive for *HP* [4]. Symptoms of patients diagnosed with *HP*‐associated dyspepsia are treated by *HP* eradication [5]. Moreover, patients undergoing endoscopy for suspected organic dyspepsia are often diagnosed with pathological findings that may be responsible for the symptoms, such as peptic ulcer, gastritis, or many other gastrointestinal or systemic causes [6].

Patients in whom no identifiable explanation for the symptoms can be detected by traditional diagnostic procedures are diagnosed as being affected by FD. A recent global survey investigating the worldwide prevalence of dyspepsia showed that FD was the most prevalent gastroduodenal disorder, with a pooled prevalence rate between 4.8% and 7.2% [7]. Functional dyspepsia can be further classified into two subtypes: postprandial distress syndrome (PDS), which is characterized by postprandial fullness and early satiety, and epigastric pain syndrome (EPS), which is characterized by epigastric pain and epigastric burning [8]. Putative pathophysiological mechanisms underlying FD are dysregulation of the gut–brain axis and immune system dysfunction, delayed gastric emptying, impaired gastric accommodation to a meal, visceral hypersensitivity to gastric distension, altered duodenal sensitivity to lipids or acids, altered antro‐duodenojejunal motility and gastric electrical rhythm, and unsuppressed postprandial phasic contractility in the proximal stomach [9]. Pathogenetic factors in FD are genetic predisposition, infection from HP or other organisms, inflammation, and psychosocial factors [9].

The current management of uninvestigated dyspepsia mostly includes general measures, acid‐suppressive drugs, and prokinetic agents. First and subsequent lines of therapy for HP‐related dyspepsia, instead, rely on a course of antibiotics based on local patterns of resistance, followed by the confirmation of HP eradication [5]. Once FD is diagnosed after ruling out *HP* infection and organic diseases, treatment primarily involves acid‐suppressive therapy, prokinetics, fundus‐relaxing agents, antidepressants, and psychological interventions, especially if these options have not been previously attempted [10]. Recent evidence suggests that microbiota imbalance is involved in the development of FD [11, 12]. Therefore, modulating the gut microbiota through the use of probiotics could represent an effective further therapeutic strategy. Probiotics are defined according to the World Health Organization as live microorganisms which, if administered in adequate amounts, can confer health benefits to the host [13]. Probiotics are able to escape the effects of gastric acid, bile, and pancreatic juice, thus colonizing the host's gastrointestinal tract and rebalance the intestinal microbiota [13]. This review of the literature aims to summarize and discuss the current evidence on the use of probiotics in the treatment of dyspepsia.

## 2 Probiotics and *Helicobacter pylori*

*Helicobacter pylori* infection provokes chronic mucosal inflammation in the stomach and duodenum, which, in turn, might lead to gastroduodenal motor and sensory dysfunction, and it has also been linked with dyspeptic symptoms [14] Its eradication is essential to solve the related symptoms, but probiotics have been shown to play a role. Since there has been a decrease in eradication rates due to increasing antibiotic resistance and more frequent side effects due to the addition of several antibiotics for a prolonged time [5] the use of probiotics has been studied together with alternative therapies. The first study on probiotics and *HP* was carried out in 1995, highlighting that *Lactobacillus acidophilus* and one strain of *Lactobacillus rhamnosus* were found to have in vitro inhibitory activities on *HP* growth [15].

Subsequently, several studies explored the role of probiotics as an additional therapy for *HP* infection [5, 16, 17, 18]. There is an open debate on the real impact of probiotic use in *HP* treatment in clinical practice, advising their use only for specific strains supported by a previously demonstrated clinical efficacy for both reducing gastrointestinal adverse events caused by antibiotics and improving the HP eradication rate. These effects seem to be achieved through enhancing compliance with the therapy [16, 17, 19]. Two recent meta‐analyses [16, 19] reported significantly increased eradication rates (Odds ratio (OR) 2.07; 95% confidence interval (CI), 1.40–3.06) and reduced side effect rates (OR 0.31; 95% CI, 0.12–0.79) during concomitant probiotic treatment to antibiotics therapy for *HP* eradication. Of note, the evaluation of the microbial shifts after *HP* treatment highlighted variations in the gut microbiome of *Bacteroides*, *Bifidobacterium*, *Clostridium*, *Enterobacteriaceae*, and *Lactobacillus* [20] strains. Since this emerging evidence suggests that not all probiotics are useful against *HP* infection and only a few strains seem to play a positive role in eradication treatment, the most investigated probiotic strains in this setting were the *Lactobacillus* and *Bifidobacterium* species [21]. However, there are still many questions to be answered on the role of probiotic supplementation in *HP* treatments, including the optimal dose and the duration of the therapy.

### 2.1 Rationale for the Use of Probiotics During *H. pylori* Therapy

Probiotics act as antagonists to *HP* by food and binding sites competition [22]. Indeed, a previous ex vivo study demonstrated reduced colonization ability by *HP* according to the evaluation of rectal swabs, stomach homogenates, and luminal contents from ileum and colon in germ‐free mice previously colonized by probiotics ( *L. rhamnosus* , strain R0011, and *L. acidophilus* , strain R0052) [23]. Notably, in another study, certain probiotic strains such as *Saccharomyces boulardii* showed interference with *HP* adhesion to gastric and duodenal cells due to its neuraminidase activity [24] selective for a(2–3)‐linked sialic acid, able to remove surface a(2–3)‐linked sialic acid, which is the ligand for the sialic acid‐binding *HP* adhesin. Moreover, probiotics can enhance mucus production by increasing the expression of MUC2 and MUC3 as shown for *L. plantarum* and *L. rhamnosus* strains [25] and epithelial junction proteins production [26]. These effects, together with the secretion of bioactive molecules and the production of IgA acting against pathogens, can strengthen the gastric mucosal layer and increase the resistance to *HP* colonization [25]. Moreover, probiotics themselves secrete antimicrobial agents derived from fermentation, such as acetic and lactic acid, which are able to lower the intragastric pH and inhibit urease activity [27].

### 2.2 Evidence for the Use of Probiotics Alone for *H. pylori* Eradication

Few studies evaluated the effect of probiotics alone in the eradication of HP, all using different probiotic strains, and only a few of them included an adequate sample size [28, 29, 30, 31]. Moreover, most of them were conducted on children [32] since the use of triple therapy in pediatric patients is associated with overall lower efficacy and increased risk of antibiotic resistance in this population [33]. The first study [34] in adults evaluating the role of probiotics ( *L. acidophilus* La1) in combination with proton pump inhibitor (PPI) therapy failed to achieve eradication of *HP* and reported only a diminished bacterial load after treatment.

Several meta‐analyses aiming at the evaluation of the role of probiotics in *HP* eradication [16, 35, 36, 37] concluded that although probiotic therapy reduces the *HP* bacterial load, this evidence does not justify their use as monotherapy. Indeed, a recent meta‐analysis on probiotic monotherapy [38], including 11 studies and 403 patients, reported a mean eradication rate of 14%, with minimal variations across different strains (i.e., 16% for *Lactobacilli*, 12% for *Saccharomyces boulardii* and 14% for multi‐strain combinations).

### 2.3 Probiotics Concomitant Treatment With Antibiotics

Several studies and meta‐analyses [16, 19, 35, 36, 37, 39, 40, 41, 42, 43, 44, 45] evaluated the adjuvant role of probiotics in addition to antibiotic therapies for *HP* (Table 1). Most of them evaluated the role of probiotics as a whole without distinguishing among differential bacterial strains or antibiotic regimens [19, 39]. Various regimens have been proposed over the years as first‐ and second‐line therapy for *HP*.

**TABLE 1:** Summary of meta‐analysis on the effect of probiotics in the eradication of *H. pylori* .

Author, year	Trials (patients)	Age	*H. pylori* eradicating regimens	Probiotics	Results
Tong 2006	14 RCTs (*n* = 1671)	Any age	Triple therapy	Several probiotic strategies	Pooled eradication rates with and without probiotics 83.6% (95% CI 80.5%–86.7%) and 74.8% (95% CI 71.1%–78.5%). OR 1.84 (95% CI 1.34–2.54)
Zou 2009	8 RCTs (*n* = 1372)	Any age	Triple therapy	*Lactobacillus*	Pooled eradication rates with and without probiotics were 82.26% (95% CI = 78.01%–86.51%) and 76.97% (95% CI = 73.11%–80.83%) OR 1.78 (95% CI = 1.21–2.62)
Szajewska 2010	5 RCTs (*n* = 1307)	Any age	Triple therapy	*Saccharomyces boulardii*	RR 1.13, 95% CI 1.05–1.21
Zheng 2013	9 RCTs (*n* = 1163)	Any age	Triple and sequential therapy	*Lactobacillus*	RR 1.14, 95% CI 1.06–1.22
Wang 2013	10 RCTs (*n* = 1469)	Any age	Triple therapy	*Lactobacillus*‐ and *Bifidobacterium*‐containing probiotics	OR 2.066, 95% CI 1.398–3.055
Li 2014	7 RCTs (*n* = 508)	Children	Triple therapy	Several probiotic strategies	Probiotic use ITT OR 1.96 (95% CI 1.28–3.02) PP OR 2.25 (95% CI 1.41–3.57)
Dang 2014	33 RCTs (*n* = 4459)	Any age	First line treatment (triple, sequential, quadruple therapy)	Several probiotic strategies	ITT RR 1.122, 95% CI 1.086–1.159 PP RR 1.114, 95% CI 1.070–1.159
Zhu 2014	14 RCTs (*n* = 2259)	Adults	Triple therapy	Several probiotic strategies	ITT OR 1.67 (95% CI 1.38–2.02) PP OR 1.68 (95% CI 1.35–2.08)
Zhang 2015	45 RCTs (*n* = 6997)	Any age	Any eradicating regimens	Several probiotic strategies	Eradication rates with and without probiotics were 82.31% and 72.08%. PP RR 1.11, 95% CI: 1.08–1.15, ITT RR 1.13, 95% CI: 1.10–1.16
Gong 2015	23 RCTs (*n* = 3900)	Subjects > 14 years old	Triple therapy	Several probiotic strategies	Pooled eradication rates without and with probiotics were 1464/2026 (72.26%; 95% CI, 67.66%–74.13%) and 1513/1874 (80.74%; 95% CI, 74.68%–82.76%). OR = 0.58; 95% CI, 0.50–0.68.
Lv 2015	21 RCTs (*n* = 3814)	Any age	Triple therapy	*Lactobacillus*, *Bifidobacterium*, *Saccharomyces* or a mixture of the three	The pooled eradication rates of the probiotic group were 80.3% (1.709/2.128) by ITT and 83.8% (1.709/2.039) by PP analyses. ITT RR 1.12, 95% CI 1.06–1.19.
Lu 2016	13 RCTs (*n* = 2306)	Adults	Any eradicating regimens	Several probiotic strategies	Probiotic use RR 1.15, 95% CI 1.10–1.20
Lau 2016	30 RCTs (*n* = 4515)	Any age	Triple therapy	Several probiotic strategies	RR 1.122, 95% CI 1.091–1.153
Lu 2016	21 RCTs (*n* = 3520)	Adults	Triple therapy	Several probiotic strategies	ITT OR 1.21, 95% CI: 0.86 1.69 PP OR 1.28, 95% CI: 0.88, 1.86
McFarland 2016	19 RCTs (*n* = 2730)	Any age	Double, triple, or quadruple therapy	Multi‐strain probiotics (mixture of 2 or more different strains of bacteria or fungi)	RR 1.12, 95% CI 1.08–1.17
Wang 2017	140 studies (*n* = 20,215)	Adults	Triple, sequential, or quadruple therapy	10 probiotic strategies	Eradication rate 84.1% in probiotic group versus 70.5% in control.
Wen 2017	17 RCTs (*n* = 1932)	Children	14‐days triple therapy	Several probiotics strategies	Probiotic use RR: 1.16, 95% CI: 1.07–1.26
Feng 2017	29 studies (*n* = 3122)	Children	Triple therapy	17 probiotic regimens	Probiotic use RR 1.19, 95% CI 1.13–1.25
Losurdo 2018	11 studies (*n* = 517)	Any age	None	*Lactobacillus*‐containing probiotics, only 2 studies *Saccharomyces boulardii* and 3 multi‐strain probiotics	Mean weighted eradication rate 14%, 95% CI: 2%–25%
Shi 2019	40 studies (*n* = 8924)	Adults	First line treatment (triple, sequential, quadruple therapy)	Several probiotic strategies	Probiotic use RR 1.140, 95% CI 1.101–1.180
Fang 2019	5 RCTs (*n* = 484)	Children	Triple therapy	*Lactobacillus*	Lactobacillus use RR 1.19, 95% CI 1.07–1.33
Zhou 2019	18 RCTs (*n* = 3592)	Any age	First line treatment (triple, sequential, quadruple therapy)	*Saccharomyces boulardii*	RR 1.09, 95% CI: 1.05–1.13
Yu 2019	11 RCTs (*n* = 724)	Any age	Triple therapy	*Lactobacillus*	RR 1.16, 95% CI 1.08–1.25
Wang 2023	34 RCTs (*n* = 9004)	Any age	Triple therapy	10 probiotic strategies (triple therapy alone, triple therapy added with *Bacillus*, *Lactobacillus*, *Saccharomyces*, *Bifidobacterium‐Lactobacillus*, *Bacillus‐Streptococcus*, *Lactobacillus‐Streptococcus*, *Lactobacillus‐Propionibacterium*, *Bifidobacterium‐Lactobacillus‐Streptococcus*, *Bifidobacterium‐Lactobacillus‐ Saccharomyces*)	Improvement in eradication rate (RR 1.14, 95% CI: 1.07–1.21, *p* < 0.01), reduction of the side effects rate (RR 0.61, 95% CI: 0.53–0.71, *p* < 0.01) *Bifidobacterium‐Lactobacillus* and *Bifidobacterium‐Lactobacillus‐Saccharomyces* had the best comprehensive performance
Musazadeh 2023	18 meta‐analyses (*n* = 47,278)	Any age	Triple and quadruple therapy	Several probiotic strategies	Pooled ES_RR_: 1.13; 95% CI: 1.11, 1.14, *p* < 0.01 ES_OR_ = 1.86, 95% CI: 1.70, 2.03, *p* < 0.01

Regarding the combination with triple therapy, a recent meta‐analysis [16] including 140 studies on adults and a total of 20,215 patients evaluated the role of probiotics in addition to triple therapy of different durations. Taken together, antibiotic regimens irrespectively to the line of treatment, the supplementation of probiotics led to higher eradication rates compared to the control group, 84.1 versus 70.5, respectively (Risk ratio (RR) 1.17, 95% CI 1.15–1.18); the authors additionally reported that *Lactobacillus acidophilus*, *Saccharomyces boulardii*, *Lactobacillus + Bifidobacterium + Enterococcus*, and *Lactobacillus + Bifidobacterium +* *Bacillus cereus* showed higher efficacy in increasing eradication rates when added to 10‐day triple therapy, whereas only the first 3 mentioned probiotic mixtures showed a benefit in 14‐day triple therapy.

As regards quadruple therapy and probiotics supplementation, a network meta‐analysis carried out in 2019 [46], including 40 studies with 8924 patients and comparing the addition of probiotics to triple and bismuth‐containing quadruple therapy, showed that the addition of probiotics was able to increase the eradication rates in all settings (RR range 1.74 for quadruple with and without probiotics to 6.08 for quadruple with probiotics compared to triple therapy), except when triple therapy plus probiotics was compared to quadruple therapy alone.

Only one trial reported data on triple therapy with esomeprazole, levofloxacin, and amoxicillin with *L. reuteri* for 14 days and without probiotic supplementation for *HP* eradication [47]. The eradication rate was significantly influenced by probiotic supplementation with *L. reuteri* (80% vs. 62%; *p* < 0.05), and the incidence of side effects was significantly lower. Moxifloxacin‐based regimens were investigated in addition to probiotics in two studies without reaching significantly higher eradication rates [48, 49].

A recent umbrella meta‐analysis published in 2023 involving 18 eligible studies revealed that probiotics have beneficial impacts on *HP* eradication (RR 1.13, 95% CI: 1.11–1.14, *p* < 0.01, and OR 1.86, 95% CI 1.70–2.03, *p* < 0.01). Greater effects on *HP* eradication were observed when higher doses (∼10^10^ CFU) and mixed strains were supplemented [50].

Comparable results were shown in a network meta‐analysis on probiotics supplementation in standard triple therapy including 9004 patients randomized to 10 kinds of therapies; when probiotics were added, the authors found better outcomes than triple therapy alone, with higher eradication rates [51].

### 2.4 Novel *H. pylori* Eradicating Therapies and Probiotics Formulations

Novel eradicating therapies have been used in combination with probiotic supplementation. Vonoprazan is a novel potassium‐competitive acid inhibitor currently approved for administration in Japan, with advantages over traditional PPIs in acid suppression in terms of rapid efficacy and long‐lasting effect duration [52]. Kakiuchi et al. [53] evaluated the additional effect of a multiple antibiotic‐resistant lactic acid bacteria preparation of *Enterococcus faecium* 129 BIO 3B‐R, in addition to triple therapy using Vonoprazan. The authors found that the eradication rate in the two groups was comparable (83.9% and 94.1%), without any significant difference either for the incidence rate of diarrhea (73.1% vs. 56.5% respectively; *p* = 0.361) or for stool consistency.

Probiotic supplementation is also achievable with other products beyond capsule/sachet‐based bacteria‐only preparations (CBOP). For example, fermented milk‐based probiotics differ from CBOP for various components able to exert a greater inhibitory effect on the HP in vivo and in vitro. A systematic review with pooled data analysis [54] including 10 RCTs and 963 patients concluded that fermented milk‐based probiotic preparations improved HP eradication by 5%–15%, without significantly influencing the rate of adverse effects.

Promising results have also been reported for other fermented foods such as Kefir [55], *Glycyrrhiza glabra* (liquorice) [56] and Thai Fermented Rice Noodles [57], which have been shown to produce bacteriocin‐like substances inhibiting *HP*.

## 3 Functional Dyspepsia

### 3.1 Rationale for the Use of Probiotics in Functional Dyspepsia

**FIGURE 1:**
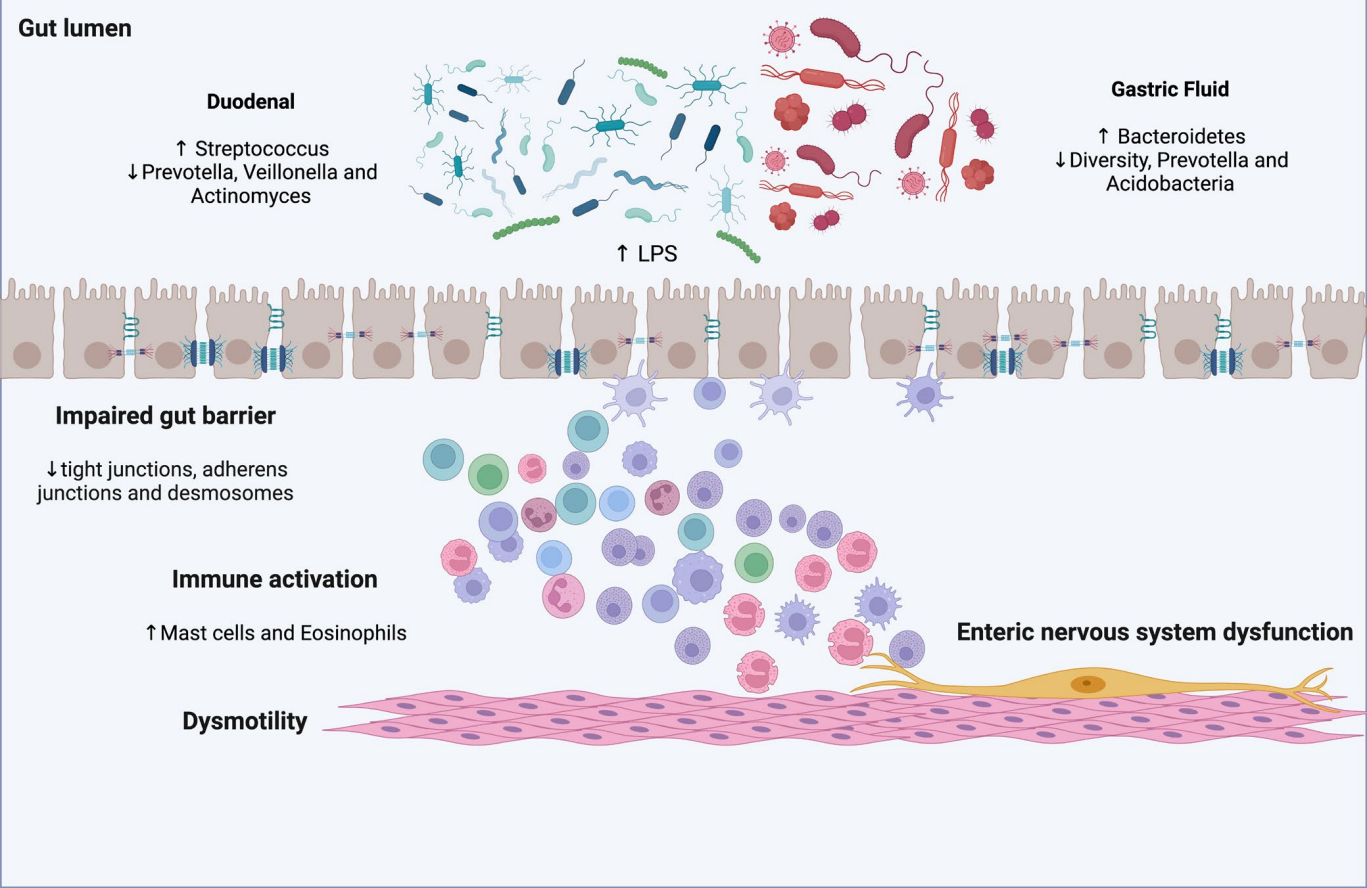
Putative pathophysiological mechanisms underlying the development of functional dyspepsia.

Definitive data about microbiota and FD are not available. Duodenal low‐grade inflammation may be involved in the etiopathogenesis of FD, inducing mucosal immune activation, duodenal barrier dysfunction, and sensory‐motor dysfunction (Figure 1). An altered duodenal microbiota, food antigens, or infection may precipitate duodenal microinflammation in a subset of FD patients [58]. Several authors analyzed FD patients' microbiota in order to evaluate its alpha diversity and the main species associated with dyspeptic symptoms. Fukui et al. showed that the relative abundance of mucosa‐associated *Streptococcus* was positively correlated with upper gastrointestinal symptoms in FD patients [59]. In this line, Zhong et al. evaluated stool and duodenal microbiota in FD patients and healthy controls. The results showed an inverse relationship between the relative abundance of *Streptococcus* and the anaerobic genera *Prevotella*, *Veillonella*, and *Actinomyces*, which were significantly decreased in patients with FD [60]. Other studies directly assessing duodenal microbiota [61, 62, 63, 64, 65] confirmed that patients with FD had lower intra‐ and interspecies variability. Among others, increased relative abundances of bacteria from the *Streptococcus* genus were the most consistently reported alteration (Table 2). Other studies assessing microbiota in gastric fluid aspirate confirmed a significantly low average interindividual diversity and a significantly low abundance of the genus *Prevotella* in patients with FD, which, therefore, was considered to be involved in its pathogenesis. These changes were also restored by treatment with yogurt containing 10^9^ colony‐forming units of *Lactobacillus gasseri* [66]. In addition, the same research group found other alterations in the GF microbiota of FD patients. The GF microbiota had a greater *Bacteroidetes* abundance than *Proteobacteria* and an absence of *Acidobacteria* in the FD group. In contrast, the GF microbiota of the HC group had a greater *Proteobacteria* abundance than *Bacteroidetes* and the presence of *Acidobacteria* [67]. They also revealed that probiotic therapy with *Lactobacillus gasseri* in patients with FD shifted the composition of the GF microbiota to the one observed in the HC volunteers [67]. Since changes in the GF microbiota paralleled the improvement of symptoms, they were suggested to be involved in the pathophysiology underlying FD.

**TABLE 2:** Summary of duodenal microbiota variations in FD.

Citation	Year	Microbial alteration	Duodenal microbiota assessment	Enrolled population
Zhong et al. [60]	2017	Increased relative abundance of *Streptococcus*. Decreased relative abundance of *Prevotella*, *Veillonella* and *Actinomyces*.	Duodenal biopsies	9 FD vs. 9 healthy controls
Fukui et al. [59]	*2020*	*Streptococcus* abundance positively correlated with severe upper gastrointestinal symptoms. Reduced β‐diversity. Increased relative abundance of *Firmicutes*	Mucosal brush sample of all sites in upper gut	11 FD vs. 7 healthy controls
Wauters et al. [61]	2021	Lower relative abundance of *Neisseria* and *Porphyromonas*	Mucosal brush samples of duodenum	28 FD vs. 30 healthy controls
Zheng et al. [64]	2022	Differences in ACE index, Shannon index and observed‐species index. Increased relative abundance in *Alloprevotella*, *Peptostreptococcus*, *Sutterella*, *Corynebacteriurn*, *Faecalibacterium*, *Staphylococcus*, *Eubacteriumnodatumgro*‐*up*, *Lachnoclostridiurn* and *Lautropia* Lower relative abundance of *Catonella*	Mucosal brush samples of duodenum	20 FD vs. 5 healthy control
		Higher activity of ureolysis and fumarate respiration in FD		
Shanahan et al. [62]	2023	Relative abundances of predominant members of the Firmicutes, Bacteroidota and Fusobacteriota phyla were linked to symptom burden in FD.	Duodenal biopsies	56 FD vs. 30 healthy controls
		Inverse relationships between the relative abundances of *Streptococcus* and *Prevotella*, and *Veillonella* spp. with gastric emptying time, were also observed.		
Tziatzios et al. [65]	2024	Lower ɑ‐diversity in FD and IBS vs. healthy controls	Duodenal aspirate	20 FD vs. 20 IBS vs. 10 healthy controls
		Relative abundance of *Chloroflexota*, *Rhodothermota* and *Thermotogota* phyla were consistently lower in subjects with FD when compared to CG but similar to IBS		
Kim et al. [63]	2024	Increased relative abundance of duodenal *Streptococcus* and reductions in stool *Butyricicoccus*	Duodenal biopsy and brushing	12 FD vs. 16 healthy controls

Also, dysbiosis in FD may be linked to impaired intestinal permeability. Indeed, indirect evidence regarding the potential physio‐pathological role of microbiota imbalances in FD comes from Vanheel et al., which reported an impaired intestinal barrier function in FD [68]. Duodenal biopsy specimens obtained from 15 patients with FD showed lower transepithelial electrical resistance (TEER) and increased paracellular passage compared with healthy controls, which is indicative of impaired mucosal integrity. In addition, abnormal expression of cell‐to‐cell adhesion proteins at the level of tight junctions, adherens junctions, and desmosomes was shown. Furthermore, an increased infiltration of mucosal mast cells and eosinophils showed the presence of low‐grade inflammation, whose severity was related to the extent of increased permeability [68]. As a possible underlying mechanism, it is supposed that the toxic bacterial cellular components of the gut, such as lipopolysaccharides, could stimulate leukocytes to produce pro‐inflammatory cytokines, triggering gastric inflammation and consequently increasing mucosal permeability, which may lead to gastric (enteric) nervous system dysfunction [69].

A recent study summarizes all the previous findings confirming the presence of duodenal mucosal inflammation and impaired expression of tight junction proteins in patients with FD. Moreover, the relative abundance of duodenal *Streptococcus* and reductions in stool *Butyricicoccus* were confirmed. These changes in the microbiota were both correlated with symptom severity. Therefore, the authors concluded that assessing changes in the abundance of stool *Butyricicoccus* may be an effective biomarker for enhancing FD diagnosis and monitoring [63].

### 3.2 Probiotic Effect in Functional Dyspepsia

Only a few studies have investigated probiotics' effects on FD (Table 3). In a preliminary randomized controlled trial (RCT) by Kim et al., 72 patients with functional gastrointestinal disorders, including FD, were assigned to one of five treatment groups containing probiotics or placebo, aiming to evaluate the efficacy and safety of commonly used probiotics. Results showed that the combination of probiotics had the most consistent pattern of gastrointestinal symptoms improvement assessed through the Gastrointestinal Quality of Life Index (GIQLI) and GI visual analogue scale (GIVAS), even if not statistically significant. The absence of conclusive evidence in this pilot trial was thought to be due to the small sample size [70].

**TABLE 3:** Summary of studies evaluating the effect of probiotics in FD.

Study, year	Country	Method	Sample size, number of patients (% female)	Age (years)	Indication	Main findings	Criteria used to define symptom improvement following therapy
Kim L. et al. 2006	USA	IMPJ I: Probiotics^a^ ( *Lactobacillus acidophilus* , *Bifidobacterium bifidum* , *Bacillus subtilis* , *Lactobacillus bulgaricus* , *Lactobacillus lactis* , and *Bacillus lichenformis*), Barley grass and oat grass juice and ionic plant‐based minerals. IMPJ II: Probiotics^b^ ( *Lactobacillus acidophilus* , *Bifidobacterium bifidum* , *Lactobacillus bulgaricus* , *Lactobacillus lactis* , *Lactobacillus brevis* , *Lactobacillus caucasicus*, *Lactobacillus fermenti*, *Lactobacillus leichmannii* , *Lactobacillus casei* , *Lactobacillus plantarum*, *Lactobacillus helveticus* , and *Saccharomyces boulardii*) No spore‐forming probiotics, Barley grass and oat grass juice and ionic plant‐based minerals IM: Probiotics^a^ PJ: Barley grass and oat grass juice, Ionic plant‐based minerals IMA: Probiotics^c^ ( *Bacillus coagulans* , *Saccharomyces boulardii*, *Bacillus subtilis* , *Lactobacillus salivarius* , and *Lactobacillus plantarum*), mushrooms and algae Placebo *12 weeks treatment*	72 (66.7%–75.0% females)	Range 21–72 years	FGID including FD (Rome II criteria)	No significant differences among groups. Consistent pattern of GI symptoms improvement in the IMPJ I and IMPJ II groups. Less consistent changes in the IM, PJ and IMA groups.	Gastrointestinal Quality of Life Index (GIQLI) and GI visual analogue scale (GIVAS)
Ianiro G. et al. 2013	Italy	Extra‐virgin olive oil enriched with anti‐oxidants (Oo/Ao) or probiotics (Oo/Pr) Oo/Ao: Two vials daily, each containing: Selenium methionine, Q10 coenzyme, ascorbyl palmitate, resveratrol, silicon dioxide. Oo/Pr: *L. reuterii* 100 billion/g, *L. rhamnosus* GG 350 billion/g, *Saccharomyces boulardii* 20 billion/g, vitamin B6 hydrochloride, inositol, silica; Q10 coenzyme. *7 days treatment*	8	—	FD (Rome III criteria)	Amelioration of nausea and abdominal pain/discomfort in subjects receiving Oo/Pr compared to Oo or Oo/Ao (*p* = 0.04)	Validated questionnaire to evaluate GI symptoms
Nakae H. et al. 2016	Japan	118 g of yogurt containing 10^9^ colony‐forming units of *L. gasseri* (LG21 yogurt) every day *12 weeks treatment*	44	Range 20–60 years	FD (Rome III criteria)	Amelioration of EPS and PDS symptoms (*p* < 0.001). Abundance of *Prevotella* significantly correlated with PDS symptoms.	Validated questionnaire
Igarashi M. et al. 2017	Japan	118 g of yogurt containing 10^9^ colony‐forming units of *L. gasseri* (LG21 yogurt) every day *12 weeks treatment*	24 21 healthy controls	—	FD (Rome III criteria)	Probiotic therapy in patients with FD shifted the composition of the GF microbiota to that observed in the HC	Validated questionnaire
Ohtsu T. et al. 2017	Japan	*L. gasseri* group: 10^9^ CFU of *L. gasseri* OLL2716/unit (85 g) of yogurt Placebo group: one unit (85 g) of yogurt made from a mixture of raw milk, dairy products, sugar, a sweetener (stevia), and raw water, fermented with *Lactobacillus delbrueckii* subsp. *bulgaricus* and *Streptococcus thermophiles*. *12 weeks treatment*	58 (75% females) patients in the *L. gasseri* group and 58 (74% females) in the placebo group	Mean age 42.8 ± 9.0 years	FD (Rome III criteria)	Not statistically significant amelioration of symptoms in the *L. gasseri* group	Validated questionnaire (4, 8, and 12 weeks assessment).
Rahmani P. et al. 2020	Iran	Placebo *L. reuteri* 10^8^ CFU *4 weeks treatment*	125	Average 7.3 ± 1.7 years old (case group) 7.7 ± 2.1 years old (control group)	FD (Rome III criteria)	68% children treated with probiotic showed a significant (*p* < 0.001) response to probiotic treatment compared to placebo group in terms of duration of pain, severity, and frequency	Wang‐Baker FACES Pain Rating Scale (WBFPRS)
Sun E. et al. 2021	China	Beverage containing 5 × 10^8^ cfu/mL of *Lactobacillus paracasei* *28 days treatment*	26 (57% female)	Mean age 44.3 ± 11.7 years	FD (Rome IV criteria)	Clinical symptom scores significantly decreased. ↑ of probiotics and relevant beneficial intestinal metabolites ↓ of harmful bacteria and intestinal metabolites	Validated questionnaire
Wauters L. et al. 2021	Belgium	Spore‐forming probiotics group: *Bacillus coagulans* MY01 and *Bacillus subtilis* MY02, 2·5 × 10^9^ CFU per capsule, twice daily Placebo group: 350 mg maltodextrin per capsule, twice daily *8 weeks treatment*	68 (32 probiotics, 36 placebo, 75% female)	Mean age 40.1 ± 14.4 years	FD (Rome IV criteria)	Decrease in PDS score ≥ 0·7 was higher for probiotics than placebo (48% vs. 20%)	Leuven Postprandial Distress Scale (LPDS), patient assessment of upper gastrointestinal disorders symptom severity index (PAGI‐SYM) and quality of life (PAGI–QOL)
Drago L. et al. 2021	Italy	*L. rhamnosus* , *L. pentosus* , *L. plantarum* and *L. delbrueckii* > 10^8^ CFU/Active Fluorescence Units [AFU] *30 days treatment*	2676 (1357 PDS patients, 1319 EPS patients)	—	FD (Rome IV criteria)	Postprandial filling and early satiety improved after treatment. No difference in the EPS group.	Validated questionnaire
Zhang Q. et al. 2024	China	Placebo (maltodextrin 2 g/day) Only PPI treatment (rabeprazole 10 mg/day) Low‐dose (1 × 10^10^ CFU/day) probiotic *Bifidobacterium animalis* subsp. *lactis* BL‐99 (BL‐99) High‐dose (5 × 10^10^ CFU/day) probiotic *Bifidobacterium animalis* subsp. *lactis* BL‐99 (BL‐99)	200	Range 18–60 years old	FD (Rome IV criteria)	CRR in FD score for the BL‐99_high group significantly higher than placebo (90% vs. 58%), than BL‐99_low (74.0%) and PPI group (70.0%)	Clinical response rate (CRR) of FD score

^a^ Fifty million CFU (six species): *Lactobacillus acidophilus*, *Bifidobacterium bifidum*, *Bacillus subtilis*, *Lactobacillus bulgaricus*, *Lactobacillus lactis*, and *Bacillus lichenformis*.

^b^ Fifty million CFU (twelve species): *Lactobacillus acidophilus*, *Bifidobacterium bifidum*, *Lactobacillus bulgaricus*, *Lactobacillus lactis*, *Lactobacillus brevis*, *Lactobacillus caucasicus*, *Lactobacillus fermenti*, *Lactobacillus leichmannii*, *Lactobacillus caseii*, *Lactobacillus plantarum*, *Lactobacillus helveticus*, and *Saccharomyces boulardii*. No spore‐forming probiotics.

^c^ Fifty million CFU (five species): *Bacillus coagulans*, *Saccharomyces boulardii*, *Bacillus subtilis*, *Lactobacillus salivarius*, and Lactobacillus.

Also, *L. gasseri* showed promising results in ameliorating symptoms in FD patients [66, 67]. A RCT with *L. gasseri* OLL2716 in 116 patients with FD showed that symptom resolution was achieved in 17% and 35.5% in the placebo and *L. gasseri OLL2716* (*p* = 0.048) arms, respectively [71]. A different trial showed that FD symptoms were relieved in participants after 28 days of treatment with a beverage containing *Lactobacillus paracasei* LC‐37 (LC‐37), with a response rate of about 90% for abdominal pain. *Lactobacillus*, *Lactococcus*, and *Weissella* significantly increased, and the abundance of harmful bacteria such as *Lachnoclostridium* significantly decreased [72]. However, results were severely limited by the open‐label design, the low number of participants, and the lack of a control group [72].

Another RCT by Wauters et al. aimed to assess spore‐forming probiotics' efficacy on reducing symptoms in functional dyspepsia as monotherapy or add‐on therapy to long‐term treatment with proton‐pump inhibitor [61]. Functional dyspepsia patients and controls received 8 weeks of treatment with probiotics ( *Bacillus coagulans* MY01 and *Bacillus subtilis* MY02, 2.5 × 10^9^ colony‐forming units per capsule) or placebo consumed twice per day, followed by an open‐label extension phase of 8 weeks. Symptoms were assessed through the Leuven Postprandial Distress Scale, along with immune activation markers (high‐sensitivity C reactive protein, lipopolysaccharide binding protein, systemic cytokines and peripheral blood mononuclear cells), and fecal microbiota. Among the 68 FD patients (50% of whom on proton‐pump inhibitors), the proportion of clinical responders was higher with probiotics than with placebo (respectively 48% vs. 20%, RR 1.95 [95% CI 1.07–4.11]; *p* = 0.028). Finally, a recent well‐conducted RCT assigned 200 FD patients to receive placebo, rabeprazole, or *Bifidobacterium animalis* subsp. *lactis BL‐99* (BL‐99; low, high doses) for 8 weeks. The primary outcome was the clinical response rate (CRR) of FD score after 8‐week treatment, which was significantly higher for the BL‐99 high dose group than that for placebo (90% vs. 58%, *p* = 0.001), BL‐99 low dose (74%, *p* = 0.044) and positive control (70%, *p* = 0.017) after 8‐week treatment. This effect was sustained until 2 weeks after treatment but disappeared 8 weeks after treatment. Metagenomic and metabolomics revealed that BL‐99 promoted the accumulation of SCFA‐producing microbiota and the increase of SCFA levels in stool and serum, which may account for the increase of serum gastrin levels, supporting its use in FD [73].

As for multistrain formulations, a probiotic combination of *Lacticaseibacillus rhamnosus* LR04, *Lactiplantibacillus pentosus* LPS01, *Lactiplantibacillus plantarum* LP01, and *Lactobacillus delbrueckii* subsp. *delbruekii* LDD01, alone or in combination with prokinetics, antacids, or proton‐pump‐inhibitors, was evaluated in FD patients to assess whether it could contribute to improving symptoms over 30 days. Patients with FD were enrolled and divided into PDS and EPS groups. All patients showed significant improvements in dyspeptic symptoms following treatment. In patients with PDS, probiotics alone resulted in the lowest prevalence of symptoms following treatment, while patients with EPS showed no clear between‐treatment differences [74].

Also, antioxidants have been proposed as a treatment for functional dyspepsia; extra‐virgin olive oil, a common ingredient of the Mediterranean diet known for its antioxidant properties, was added to the common diet of 8 subjects with functional dyspepsia for 7 days to evaluate its effect when enriched with antioxidants or probiotics (*L. reuterii*, *L. rhamnosus* GG, *Saccharomyces boulardii*). All patients had a significant improvement of dyspeptic symptoms, with a greater effect observed for the probiotic‐enriched group [75].

A systematic review included 6 RCTs, only 3 of which were included in the meta‐analysis, reported that *Lactobacillus* strains showed potential positive effects in terms of improving upper gastrointestinal symptoms in patients with FD. Probiotic supplementation showed a trend of improving the global dyspepsia score (RR: 1.35, 95% CI 0.99–1.84; *p* = 0.061) and bacterial composition in the gastrointestinal tract, without any serious adverse events. The evidence, however, is insufficient to draw clear conclusions regarding efficacy [76]. In summary, dysbiosis is one of the main pathological mechanisms in the pathogenesis of FD. The use of probiotics in FD could be beneficial, but it is still debated due to the lack of strong evidence available at present for their use.

## 4 Conclusion

Probiotics alone have no meaningful influence on the eradication of HP infection, although a significant reduction in adverse events has been reported during probiotic supplementation, especially diarrhea and abdominal discomfort, which in turn may enhance patients' compliance with the therapy and increase eradication rates. A trend toward the efficacy of probiotics in ameliorating FD symptoms is supported by several pieces of evidence. The main pitfall of studies assessing the efficacy of probiotics in FD is a high variability in the strain that can be more beneficial, in the duration of the treatment, and in the criteria used to define symptom improvement following therapy. Nevertheless, data on probiotics remain promising, and given their safety and low rate of side effects, their use should be further investigated in order to determine the optimal probiotic strain, dosage, and therapy duration.

## Author Contributions

G.M. and G.B. designed the review. G.M. and M.F. performed the literature search. All authors drafted the manuscript, critically revised it, and approved the final version of the manuscript. All authors approved the final version of the article, including the authorship list.

## Ethics Statement

Ethical review and approval were waived for this study due to the use of already available published data.

## Consent

Informed consent was obtained from all subjects involved in the study by the investigator of each published study included in the present review.

## Conflicts of Interest

The authors declare no conflicts of interest.

## Data Availability

The data presented in this study are openly available in Medline and Embase.
